# An Investigation of the Effect of Transfected Defective, Ebola Virus Genomes on Ebola Replication

**DOI:** 10.3389/fcimb.2020.00159

**Published:** 2020-04-21

**Authors:** Sophie J. Smither, Isabel Garcia-Dorival, Lin Eastaugh, James S. Findlay, Lyn M. O'Brien, Jonathan Carruthers, E. Diane Williamson, Carmen Molina-París, Julian A. Hiscox, Thomas R. Laws

**Affiliations:** ^1^CBR Division, Dstl Porton Down, Salisbury, United Kingdom; ^2^Institute of Infection and Global Health, University of Liverpool, Liverpool, United Kingdom; ^3^School of Mathematics, University of Leeds, Leeds, United Kingdom

**Keywords:** ebola, defective interfering particles, DIPs, deletion, *in vitro*

## Abstract

As the ongoing outbreak in the Democratic Republic of Congo illustrates, Ebola virus disease continues to pose a significant risk to humankind and this necessitates the continued development of therapeutic options. One option that warrants evaluation is that of defective genomes; these can potentially parasitize resources from the wild-type virus and may even be packaged for repeated co-infection cycles. Deletion and copy-back defective genomes have been identified and reported in the literature. As a crude, mixed preparation these were found to have limiting effects on cytopathology. Here we have used synthetic virology to clone and manufacture two deletion defective genomes. These genomes were tested with Ebola virus using *in vitro* cell culture and shown to inhibit viral replication; however, and against expectations, the defective genomes were not released in biologically significant numbers. We propose that EBOV might have yet unknown mechanisms to prevent parasitisation by defective interfering particles beyond the known mechanism that prevents sequential infection of the same cell. Understanding this mechanism would be necessary in any development of a defective interfering particle-based therapy.

## Introduction

Like all of the filoviridae, the Ebola virus (EBOV) genome is composed of a negative sense single stranded RNA, which is present in the cell as a ribonucleoprotein complex (RNP). During virus assembly this RNP becomes enveloped in a host-cell phospholipid membrane, by incorporating various viral membrane proteins, including the glycoprotein (Martin et al., 2018). The virus is believed to survive long term in an environmental niche that is likely to involve species of African bats (Singh et al., 2017). Occasionally the virus jumps the species barrier and causes outbreaks of Ebola virus disease (EVD) in humans, which is severe with characteristic fever, viraemia and occasionally hemorrhage (Brown et al., 2017), and a case fatality rate as high as 80% in some outbreaks (Nyakarahuka et al., 2016). The virus is transmissible between people via contact with body fluids and is therefore capable of causing epidemics of varying sizes (Brown et al., 2017). At the time of writing this article, the most serious epidemic to date occurred in West Africa in 2014; however there is currently an uncontrolled outbreak in the Democratic Republic of Congo (World Health Organization, 2018) and spreading into Uganda.

Viral defective interfering particles (DIPs) were first described in the 1950's in influenza virus (Gard et al., 1952). DIPs are viral particles capable of translocating between cells but, they contain defective genomes. The defective genomes are unable to reproduce themselves or manufacture all the proteins required for viral assembly. DIPs can, however, replicate when co-infected with wild-type virus, potentially to the detriment of the wild-type virus by sequestering cellular resources and viral proteins. In the mononegavirales (a family of viruses where genomes are encoded by a single negative strand of RNA and of which EBOV is a member), defective genomes spontaneously arise due to incorrect amplification of the genome. The first DIPs in mononegavirales were found in vesicular stomatitis virus Indiana strain (Huang and Baltimore, 1970). There are thought to be two main types of defective genome. Deletion defective genomes arise where a fusion event occurs between the 3' and 5' ends of the genome, possibly where a loop has formed. The result is a genome with (normally) large regions of coding sequence being deleted but with the 3' and 5' ends intact (Resende Rde et al., 1992). Copy-back defective genomes are believed to form when the RNA dependent RNA polymerase (RdRP) copies back onto the strand under construction. The result is a genome with the same origin of replication at both ends; one in sense and one in anti-sense. These are usually the wild-type's 5' end (in antisense form) because genomic replication is heavily biased for antisense to sense replication (Schubert and Lazzarini, 1981).

With regards to EBOV, DIPs were first described by Calain et al. (1999). In their study, Calain et al. used a serial passage at high multiplicity of infection (MOI) to enhance the probability of defective genome formation. They mapped several of these and found that, when they were co-infected with the low passage virus, cytopathology was tempered. It is difficult to say what role DIPs might play in actual human infection; however, Calain et al. later suggested that it was possible that defective genomes played a role in latent infections possibly observed in cases of EVD where there is recrudescence several months after patients are convalescent and EBOV negative in their bloods (Calain et al., 2016). It is unclear why, since DIPs are readily generated in the laboratory, and they temper viral pathology, we are unable to find reports of epidemics and disease in individuals spontaneously resolving because of DIPs. One reason might be that EBOV is known to have the ability to prevent further infection of the cell that it infects (i.e., to prevent super-infection/co-infection). This phenomenon of infection blockade is driven by the Δ-peptide (Radoshitzky et al., 2011). In the studies by Calain et al. (1999), DIPs were used at high MOI and simultaneously to the wild-type virus infection. Therefore, coinfection in these studies was significantly more probable. Conversely in epidemics, and in the host, EBOV is a budding virus (Martin et al., 2018) and this means exposure of neighboring cells will occur across a greater time period than for a bursting virus. This prevention of super-infection/co-infection is therefore likely to provide a natural defense against parasitisation by DIPs. The existence of a system of infection blockade does not however preclude the possibility that DIPs might be useful as therapies provided one of the following considerations are taken on board: (1) deliver defective genomes prior to EBOV and in a manner that does not preclude super infection (i.e., absence of Δ-peptide), (2) use a route of cellular entry that is independent of EBOV uptake, or (3) be available systemically at high titer.

Here we have explored the impact that defective genomes of EBOV might offer by considering their therapeutic potential, in *in vitro* infection studies as synthetic constructs. It is obviously not conceivable to use the high passage mixed population of virus and DIP observed by Calain et al. (1999) because of safety issues associated with an uncharacterised population containing real virus. For this reason nucleic acid synthesis and molecular biology were used to recreate and evaluate the therapeutic potential of two of the defective genomes that Calain et al. (1999) characterized.

## Materials and Methods

### Construction and Quantification of Defective Genomes

For the first deletion defective genome (hereafter referred to as DG-d1) described by Calain et al. (1999) a T7 promoter sequence was cloned along with the last 618 nt of the 5′ end of the EBOV-Makona genome and the first 373 nt of the 3′ end of the EBOV-Makona genome including the translational start codon of the NP gene (antisense strand from 5′ to 3′), a hepatitis delta virus ribozyme sequence and the T7 terminator sequence. For one of the other deletion defective genomes described by Calain et al. (1999) (hereafter referred to as DG-d2) a T7 promoter sequence was inserted along with the last 672 nt of the 5′ end of the EBOV-Makona genome and the first 155 nt of the 3′ end of the EBOV-Makona genome (Genebank Accession KJ660347.2). All plasmids preparations and synthesis were done by GeneArt™ (ThermoFisher Scientific, UK). The DIs were synthesized and then inserted into a cloning plasmid (pMK-RQ), after that the insert was cut and put into the appropriate expression plasmid (pUC 57_A338), which is the same backbone plasmids used for the EBOV min-genome system. This genome template was used so that a mini-genome system might be used as part of an initial screen (Mühlberger, 2007). The output of mini-genome-based assay broadly correlated with that of the high containment assay (data not shown).

In order to test deletion defective genomes in cell lines that do not express the T7 polymerase, for example Vero C1008 cells, RNA from the DIP plasmids was made *in vitro*. The MEGAscript® T7 transcription kit was used (Thermo Fisher, AM1333) following the instructions of the manufacturer.

The control RNA used was the pTRI-Xef control template, from the MEGAscript® T7 transcription kit, which is a linearized TRIPLEscript plasmid containing the 1.85 kb Xenopus elongation factor 1α gene under the transcriptional control of T7 promoter (pTRI-Xef 1). The size of the transcript after the *in vitro* transcription is 1.87 kb.

The DG-d1 genomes were quantified using a bespoke PCR methodology. The reverse transcription (RT) step was done using a SuperScript™ IV First-Strand Synthesis System (Thermo Fisher – 18091050) and gene-specific primers (forward = TTGAACCTGAAAACGAAAGGAGTCC, reverse = AGCCCAGACCTATCGTTAAAGC) following the manufacturer's instructions (Thermo Fisher; UK). A mixture of 1 μg of RNA from the samples was mixed with 20 μM of DG-d1 or primers, 2 μL of 10 mM dNTP mix and completed with Diethyl pyrocarbonate -treated water up to 13 μL. The mixture was then incubated at 65°C for 5 min, then cooled on ice for 1 min and 8 μl of the cDNA synthesis mix was added (4 μL of 5X Super Script IV buffer, 1 μl of DTT (100 mM), 1 μl of Ribonuclease Inhibitor, and 1 μL of SuperScript ^*TM*^ IV Reverse Transcriptase). The mixture was incubated at 55°C for 10 min and finally 1 μL of RNaseH was added to the mixture and incubated at 37°C for 20 min. To do the Real-time PCR, an iTaq™ Universal SYBR® Green Supermix (BIO-RAD, 1725120) was used following the instructions of the manufacturer. Several controls were used for this, including RNA from DG-d1 (for the RT step) and a DG-d2 plasmid with Pol1 Promoter (PCR control). The conditions for the Real-time PCR were 1 step at 95°C for 2 min and 30 steps at 95°C for 30 s, then 55°C for 30 s and 72°C for 30 s.

### Cells

Vero C1008 cells (ECACC Cat. No.85020206) were obtained from Culture Collection, Public Health England, UK. Vero C1008 cells were maintained in Dulbecco's minimum essential media supplemented with 10% (v/v) fetal calf serum, 1% (v/v) L-glutamine and 1% (v/v) penicillin/streptomycin (Sigma). For experimental purposes, the fetal calf serum concentration was reduced to 2% (v/v).

### Ebola Virus Growth and Manipulation

Ebola virus *H. sapiens*-tc/COD/1976/Yambuku-Ecran, hereafter referred to as EBOV-Ecran was used in all studies. This virus, previously known as EBOV “E718” (Kuhn et al., 2014) was supplied by Public Health England. Passage 5 material was used to infect Vero C1008 cells. Virus was harvested on day 5 post-inoculation and titrated to produce a working stock at 1 × 10^7^ TCID_50_/mL. This strain was selected because it would be able to directly transition into mouse studies should the strategy be found to be sufficiently effective to warrant further study. The disparity between viral strains used to generate the defective genomes was considered bioinformatically and, comparing the EBOV reference sequence from 1976 (NC_002549.1) with KJ660347.2, there was no nucleotide difference in the leader sequence of this virus when you compared with the EBOV Mayinga reference sequence from NCBI: NC_002549.1. However, there were few nucleotide differences in the trailer region when compare both viruses.

EBOV-Ecran was titrated in 96-well plates using the endpoint fifty percent tissue culture infectious dose (TCID_50_) assay (Smither et al., 2013). Briefly, virus was 10-fold serially diluted in 96-well plates of Vero C1008 cells. After 1 week of incubation at 37°C/5% CO_2_, all wells were observed under the microscope and scored for presence or absence of cytopathic effects. The 50% end-point was then calculated using the method of Reed and Muench (1938). RNA extractions were performed using the QiAMP Viral RNA Mini Kit (Qiagen, UK). Two 50 μL elutions were performed for each sample to increase the volume available for RT-PCR.

In order to test the effect of the defective genomes in cells infected with EBOV-Ecran the genetic material of EBOV-Ecran was quantified using the RealStar® Filovirus Screen RT-PCR Kit (Altona diagnostics, Country) following the instructions of the manufacturer. Relevant positive controls were included. We have performed this assay many times against a standard curve of plasmid containing the L gene from EBOV-Ecran (the plasmid was taken from an established mini-replicon assay Mühlberger, 2007 and the copy number was estimated from the concentration, estimated using a nanodrop and the online tool https://cels.uri.edu/gsc/cndna.html). We found that the equation *GE* (*genome equivalents*) = 2^58.37*-*1.17Ct^ can be used to estimate the number of genomes from the Ct values (Supplemental Material 1, Supplemental Figure 1). In this context “GE” might consist of incomplete negative sense RNA molecules encoding this sequence of the L gene. However, we do not believe that these will be common (<5%) based upon observations made with next generation sequencing (paper in preparation).

We found that the qRT-PCR for DG-d1 could be similarly be used to provide estimates of copy numbers where the equations is *GE* (*genome equivalents*) = 2^49.02*-*0.87 × Cq^ (Supplemental Material 1, Supplemental Figure 2). The numbers of copies in the standard curve were estimated using the nanodrop and online tool as above. Both qRT-PCR assays were performed using the BIORAD CFX Connect – Real Time System.

### Efficacy Assays

Twenty-four well plates were seeded with Vero C1008 cells at 2 × 10^5^ cells/mL (1 mL/well). EBOV-Ecran was added to the cells at an MOI = 5 and after 2 h incubation at 37°C/5% CO_2_ the supernatant was removed. Transfection was performed following the manufacturer's instructions with 2.5 μL Lipofectamine 2000® (Invitrogen) and 1 μg RNA (defective genome or control RNA, at 500 ng/μL, produced in this study) in a final volume of 500 μL Optimem Reduced Serum media (Fisher Scientific). After addition of the transfection reagents, cells were incubated for 4 h at 37°C/5% CO_2_ and then washed twice with tissue culture media and 1 mL of media was added to each well. Supernatant samples were collected and used for viral enumeration by TCID_50_ assay and RNA extractions. Samples were collected 6 h and 48 h after EBOV-Ecran infection.

### Statistical Analysis

Graphs were prepared using the software Graphpad PRISM V7.0. Statistical analyses were performed using IBM SPSS V21.0. For the correlation analysis, all data were transformed to the logarithm of 10 in order to better fit a Gaussian distribution. Both Pearson's and partial correlations were performed. To test the effect of transfection with defective genomes the ratio of virus released between control and test was found and subjected to 1 sample *T*-test.

## Results

### Design of Defective Genomes

**Figure 1:**
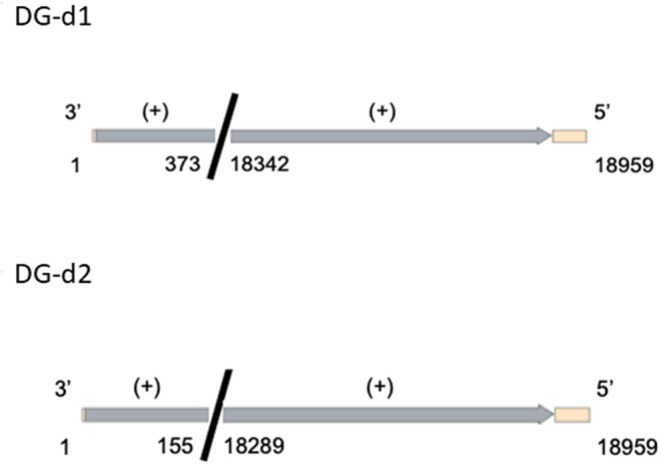
Genetic maps of the defective genome constructs from Calain et al. (1999). The Light orange region correspond to regions of the backbone plasmid (pUC 57_A338); the gray regions correspond to the virus regions that were used from the EBOV viral sequence (KJ660347.2). The colors are for guidance only.

In order to investigate the effect of defective genomes on viral biology two candidates were chosen from a study that identified and characterized DIs in cell culture. Data was used from previously published work (Calain et al., 1999) to synthetically recreate two defective genome clones DG-d1 and DG-d2 (Figure 1). The cDNA of these molecules was cloned into a plasmid backbone for amplification. The DI sequence was flanked by a T7 promoter for synthesis of RNA and a HDV ribozyme and T7 terminator to ensure near authentic 3′ and 5′ termini.

### Defective Genomes Reduce Viral Replication

In order to evaluate the potential for defective genomes to reduce viral progeny, Vero C1008 cells were infected with EBOV-Ecran. After infection, the cells were washed and transfected with the defective genome. Some wells of cells were harvested at this stage (6 h post exposure to virus and 4 h post exposure to defective genome RNA) to assess how much of virus and RNA had entered the cells. Later, after 48 h, more samples were taken from the supernatant to evaluate how much virus had been released. Under initial conditions, no evidence was found for differences in EBOV-Ecran wild-type viral uptake between cells exposed to the different RNA molecule (none, control or defective genome; when measured by qRT-PCR). The qRT-PCR assay used here targets a sequence in the L gene that is not encoded by the defective genomes. This makes cross reactivity highly unlikely. Moreover, this assay does not give signal in control groups where cells were transfected with defective genomes and but not infected by virus. A strong correlation was observed between the uptake (measured by qRT-PCR) in the non-transfected and RNA transfected controls (*P* = 0.007) indicating that variability existed extra-experimentally (Supplemental Material 2, Supplemental Figure 3). Given that a small range of viral uptake (measured by qRT-PCR) had occurred, we considered the question of whether the uptake might influence the viral output (Supplemental Figure 3). A partial correlation analysis was performed comparing uptake data to the output data (48 h later), where the analysis controlled for the treatment group effects. This analysis did not suggest that any variation in uptake influenced viral output (*R* = 0.388, *P* = 0.067).

**Figure 2:**
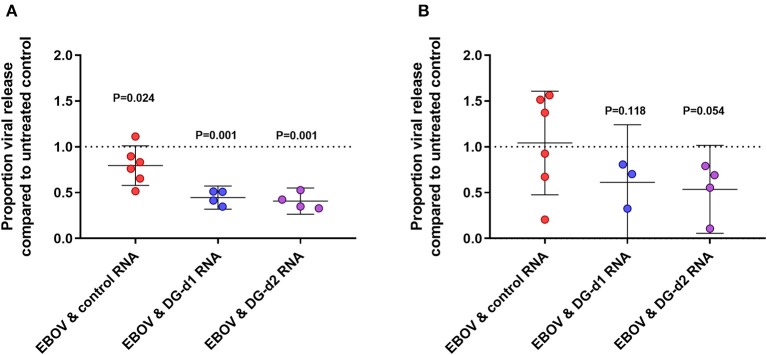
The effect of defective genomes on viral release. 2 × 10^5^ Vero c1008 cells were infected with 1 × 10^6^ TCID_50_ infectious units of EBOV strain Ecran (~1 × 10^12^ genomes) for 2 h then washed. The cells were then transfected with 1 μg of control RNA (red dots) or DG-d1 RNA (blue dots) RNA or DG-d2 RNA (purple dots) for 4 h. Un-treated Ebola virus only infection was used for comparison. The cells were then incubated and cell culture supernatants at 48 h post infection were taken and analyzed. The virus was enumerated using qRT-PCR **(A)** and TCID50 assay **(B)**. Data shown are normalized to the EBOV-Ecran only control for each experiment. One sample *T*-tests were performed to indicate the likelihood that the difference would have occurred through random chance. The *P*-values are given for the 95% level. Each data point is an independent experiment and the geometric mean of 3 replicates generated from independent wells within the same experiment.

After 48 h of growth, viral progeny was estimated in the supernatant (Figure 2A). This time point was selected because viral release occurred at this time with minimal viral particle degradation in the supernatant (data not shown). Across each experiment, the addition of either DG-d1 or DG-d2 defective genomes had reduced the numbers of viral progeny by ~50% (estimated by qRT-PCR) when compared to cells infected with just EBOV or EBOV and a non-specific control RNA). Each deletion defective genome was compared to controls 4 times for statistical rigor and this effect on release of EBOV RNA from cells was robust (*P* = 0.001, for both DG-d1 and DG-d2). The assay was of sufficient power to identify that the transfection of control RNA had a small negative effect on viral release (*P* = 0.024).

Viral release was also measured by TCID_50_ assay. The TCID_50_ data generated was generally confirmatory of the qRT-PCR data; however, we found the TCID_50_ assay had inherently more variability (Figure 2B). When compared to the transfected control we found both DG-d1 and DG-d2 reduced the TCID_50_ titres (*P* = 0.118, *P* = 0.054, respectively). The TCID_50_ broadly data supports the qRT-PCR data; however not to the same level of confidence.

### Defective Genomes Are Not Released

**Figure 3:**
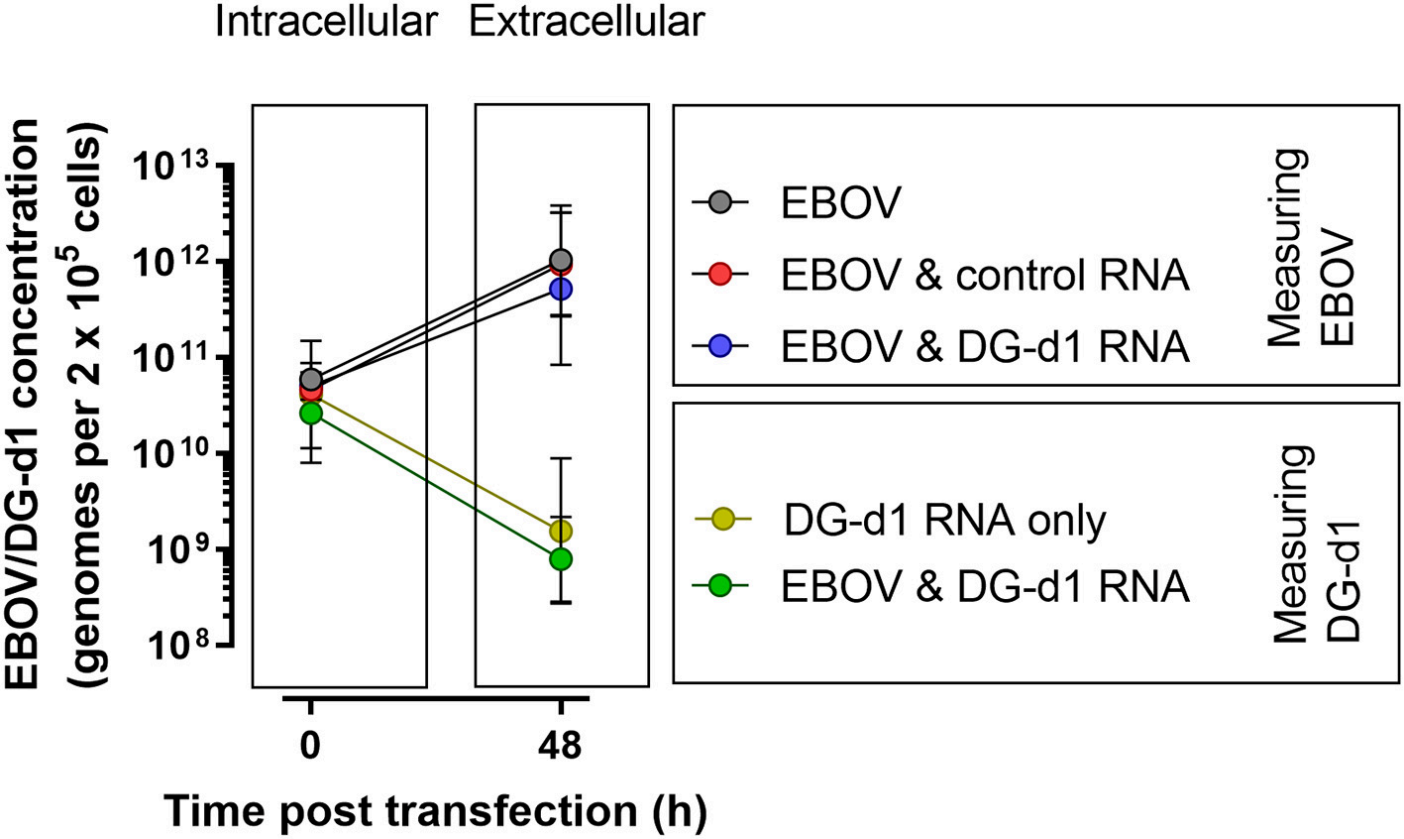
The release of defective genomes. 2 × 10^5^ Vero c1008 cells were infected with 1 × 10^6^ TCID_50_ infectious units of EBOV strain Ecran (~1 × 10^12^ genomes) for 2 h then washed. The cells were then transfected with 1 μg of DG-d1 RNA for 4 h. The cells were then incubated. Samples of the cells at time 0 and supernatant at 48 h post infection were taken. The viral genomes (defective and wild-type) were enumerated using qRT-PCR. The graph shows the count of each viral type (wild-type and DG-d1) within the cells at the onset of the experiment and the counts of viral type in the extracellular space after 48 h. Each data point is the geometric mean (± 95% confidence intervals) of 3 independent experiments, where each experiment is the geometric mean of 3 replicates generated from independent wells within the same experiment. Where EBOV Ecran was measured, black lines link the data points and the gray circles indicate non-transfected control infected cells, the red circles indicate infected cells transfected with control RNA and the blue circles indicate the infected cells transfected with DG-d1. Where the DG-d1 was measured, the green lines link the data points and the green circles indicate the infected cells transfected with DG-d1 and the yellow circles indicate uninfected cells transfected with DG-d1.

One of the potential strengths of using DIPs as a therapeutic is that they might parasitize wild type virus to propagate. In this way their pharmacokinetics may self-regulate (or self-propagate) to the requirement (the level of infection occurring). This is less feasible when considering EBOV due to the remarkable speed by which EBOV blocks “super-infection;” however, this is very much still a point of interest. We hypothesized that there are two mechanisms by which these defective genomes might interfere with viral replication. Firstly, the defective genomes might be replicated, sequestering and using resources for the replication of wild-type genomes. Secondly, the defective genomes might parasitize viral proteins. Additionally, it is possible that both of these assertions are correct. It is important to evaluate which (if either) of these are likely to be true. In order to assess the potential for the deletion defective genomes to propagate alongside the wild-type virus, a qRT-PCR method was developed where one primer recognizes only the unique fusion sequence of defective genome DG-d1. Cross-reactivity of this assay is highly unlikely. This fusion sequence does not exist in wild-type virus. Moreover, this assay does not give signal in control groups where cells were infected with wild-type virus but not transfected with defective genomes. We were not able to develop a similar assay for DG-d2. The assay was used to analyse the samples from three of the experiments shown in Figure 2. Importantly, the transfection efficiency for DG-d1 was similar to the infection efficacy. The transfection reactions had placed similar numbers of defective genomes into the cells as EBOV genomes had managed to infect (Figure 3). We found that the numbers of defective genomes in the released supernatant were several orders of magnitude lower than that of the released EBOV genomes (Figure 3). Moreover, the numbers of defective genomes in the supernatant were not different when comparing the cells that were transfected by defective genome only and cells that were co-infected by both EBOV and transfected defective genomes.

## Discussion

Two deletion defective genomes (Calain et al., 1999) were synthetically reproduced. An experimental, *in vitro*, co-infection system was developed for the analysis of the effect of defective genomes on the growth of EBOV-Ecran. The defective genomes were transfected after infection had occurred and residual virus had been washed away. This was to reduce the likelihood of artifacts in the virus replication cycle occurring because of the lipofectamine used to transfect the cells with the defective genome. Cumulative control data suggest that our experimental system is robust. Some variation is observed in the quantified viral uptake; however, this does not persist throughout the experiment. Therefore, this variation is likely to be due to slight differences in when samples were taken, rather than actual differences in initial conditions. We found that the two deletion defective genomes repeatedly reduced viral replication by ~2-fold compared to controls. This effect was most evident when viral release was measured by qRT-PCR and the number of genomes released; however, we observed some evidence that this was the case also with infectious units (measured by TCID_50_). The disparity in levels of confidence between these two measures can be explained by the difference in precision of the two assays. The TCID_50_ assay measurements are ordinal to the level of number replicate wells and the number of dilutions and the qRT-PCR is not and Ct/Cq values can be calculate to many decimal points. We acknowledge however the possibility exists that only released RNA is affected by the addition of defective genomes. Additional work should be performed to determine the function of these molecules in a dose dependent manner, in line with Koch's postulates. Indeed it is possible that more favorable ratios of MOIs may exist. Here we used one MOI of 5, which is quite high deliberately to maximize the potential for cells to be co-infected by virus and defective genome. Another way that the effect might be maximized would be to change the time of defective genome intervention in relation to viral infection. In single experiments (not shown here) we looked at transfection of defective genome RNA prior infection and close to the end of the anticipated eclipse stage but saw no effect. The work we present here has infection prior to transfection in case the transfections might be deleterious to infection.

When Calain et al. co-infected naïve cells with the diversity of DIPs and wild type virus and low passage virus, they observed a near complete cessation of cytopathology (Calain et al., 1999). The effect we observed was more modest. We also found that the RNA control also had a lesser but measurable negative effect on EBOV release (measured by qRT-PCR). This may be driven by low level immune activation. Vero cells are capable of responding to interferon, thus demonstrating their immuno-competence; however, the cells do not release interferon (Desmyter et al., 1968). There are multiple points where single stranded RNA can be recognized by the mammalian cell, reviewed in Jensen and Thomsen (2012) and it is likely that the effect observed was driven by one of these. It is unclear whether such an immune-stimulating effect would be additive for the sequences encoding the defective genomes.

We found that it is unlikely that defective genomes are being released from infected cells as particles (or otherwise) in our system. Despite this, it was still possible that the defective genomes were being replicated within the cell. This presents two possibilities: (1) defective particles were made but not released or (2) they were not made. We were only able to test the intracellular component of the assay once and saw no replication of DG-d1 RNA (data not shown); however both possibilities are possible when only a single experiment is available for evidence. If we presume that the defective genomes are not being replicated in the system, we might further propose that the mechanism of action is that defective genomes sequester protein resources. It is unclear why the defective genomes were not replicated to a significant extent in the presence of EBOV. However, understanding this phenomenon could potentially inform about how viruses are able to evade parasitisation by DIPs. Such adaptations would clearly convey a selective advantage to the virus. We might postulate that the RDRP might have some yet unknown ability to detect truncated genomes, or that the EBOV genomes are chemically modified in some way that the recombinately produced defective genomes are not and the RDRP recognizes this. However this is highly unlikely otherwise laboratory systems such as the mini-replicon would not function and they do (Garcia-Dorival et al., 2014). An alternative hypothesis is that the defective genomes were not sufficiently co-located to interact with the ribonucleoprotein (RNP). This is also not true because some restricting effect on viral release was observed where defective genomes and EBOV were placed inside cells together. Moreover, we observed high numbers of both defective genome and wild-type genome per cell. The only credible explanation these authors have been able to postulate is that the RNP has some way of prioritizing longer genomes. If the RNP is able to bind anywhere to the RNA genome and then translocate to the origin of synthesis; then, purely on the merit of its size alone, our defective genomes will associate to an RNP once for every 20 wild-type genomes. At this ratio, it is clear that the defective genomes will quickly be out competed, especially if RNPs stay associated to their genomes and are a rare resource. This is likely to be true as the L protein is that last to be transcribed during an EBOV infection of a cell (Mühlberger, 2007).

Here we only present deletion defective genome data. We did investigate the potential for copy-back variants; however we did not observe an effect (data not shown, single experiment for each). We attributed this to the palindromic nature of the copy-back genomes generating “hairpin” type structures that then caused siRNA type effects (where the host cell silences RNA using short templates of invasive RNA). These “hairpin” type structures might be recognized by the Dicer proteins and broken down into short duplexes. This would obviously have to occur prior to the establishment of EBOV VP35 suppression of Dicer co-factors (Fabozzi et al., 2011). It was our hope that recombinately produced defective EBOV genomes would have potential in reducing wild-type viral replication by reproducing in competition for resources. While an EBOV replication reducing effect was observed, this effect was limited (approximately halving) and we observed no significant release of the defective genomes. Further research into why this phenomenon has occurred might reveal novel aspects of how EBOV protects itself from parasitisation by defective genomes and possibly even the function of the RDRP.

## Data Availability Statement

The datasets generated for this study can be issued after request. DSTL/JA114401 Content includes material subject to Crown copyright (2019), Dstl. This material is licensed under the terms of the Open Government License except where otherwise stated. To view this license, visit http://www.nationalarchives.gov.uk/doc/open-government-licence/version/3/ or write to the Information Policy Team, The National Archives, Kew, London TW9 4DU, or psi@nationalarchives.gov.uk.

## Author Contributions

SS, CM-P, JH, and TL conceptualized the study and acquired the funding. TL helped with data curation. JC and TL worked on formal analysis and visualization. SS, IG-D, LE, JF, LO'B, and JH contributed to methodology. SS, JH, and TL were responsible for project administration. LE contributed to resources. SS, EW, and JH supervised the study. SS and TL wrote the original draft. SS, IG-D, JF, LO'B, EW, and TL reviewed and edited the manuscript.

## Conflict of Interest

The authors declare that the research was conducted in the absence of any commercial or financial relationships that could be construed as a potential conflict of interest.

## Supplementary Material

Two sections of supplemental material are associated with this manuscript. The first section describes the calculation used to transform data from the two qRT-PCR assays into estimates of numbers of both wild-type EBOV-Ecran and defective virus DG-d1. The second section consists of a single figure that visualized the sources of residual error on the efficacy assay.
