# Supplementary material for: An Investigation of the Effect of Transfected Defective, Ebola Virus Genomes on Ebola Replication
Source: Front Cell Infect Microbiol. 2020 Apr 21;10:159. doi: 10.3389/fcimb.2020.00159 (PMC7187655; doi:10.3389/fcimb.2020.00159)
Supplement: Supplementary file 1 [file Data_Sheet_1.PDF]

Supplementary Material 1

1 Standard curve calculation

Suppose that $V_{EBOV}(0)$ denotes the number of EBOV viral genome copies in a standard sample, and let C_t be the number of RT-PCR cycles required to reach a chosen threshold of copies, $V_{EBOV}(C_t)$. Provided that the number of copies approximately doubles with each RT-PCR cycle, these two quantities are linked by the equation

$$V_{EBOV}(C_t) = V_{EBOV}(0)2^{bC_t}$$

Here, the constant b can be interpreted as the efficacy of the RT-PCR doubling. When $b = 1$, the ratio of $V_{EBOV}(C_t)$ between subsequent cycles is 2, indicating that the number of genome copies exactly doubles with each cycle. Given that we are instead interested in identifying the number of genomes in the original sample, of greater use to us is the equation

$$V_{EBOV}(0) = V_{EBOV}(C_t)2^{-bC_t} = 2^{a-bC_t}$$

$$\log_2(V_{EBOV}(0)) = a - bC_t$$

the latter of which is the equation of a straight line. The standard curve can therefore be obtained by estimating the constants a and b from known initial copy numbers, determined by mass. Using the statistical software R to perform this linear regression yields $a = 58.37$ and $b = 1.17$, with respective p-values of 3.4×10^{-4} and 1.5×10^{-2}.

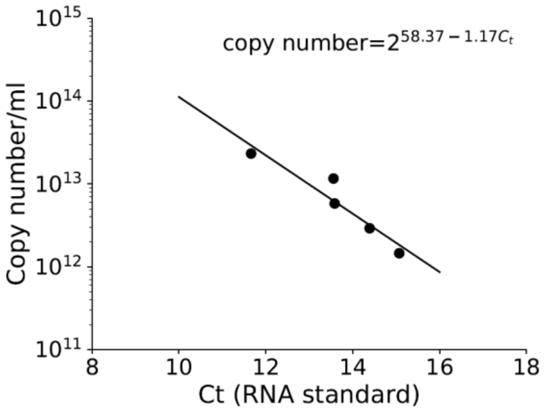

Supplemental figure 1: A depiction of this EBOV standard curve alongside the known copy numbers used to parametrise it.

In the same way that this approach has been used to construct a standard curve for the copy number of EBOV genomes, a standard curve can also be constructed for the copy number of deletion defective genomes (DG-d1). Repeating the linear regression using known copy numbers of DG-d1

gives the estimates $a_{DG} = 49.02$ and $b_{DG} = 0.87$, both of which are significant at a 0.1% significance level.

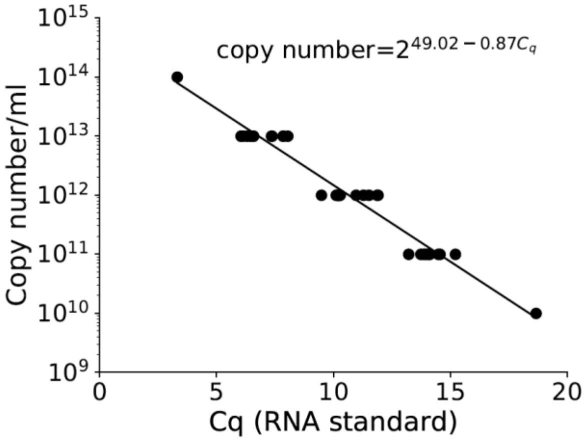

Supplemental figure 2: A depiction of this DG-d1 standard curve alongside the known copy numbers used to parametrise it.

Supplementary Material 2

2 Experimental initial conditions

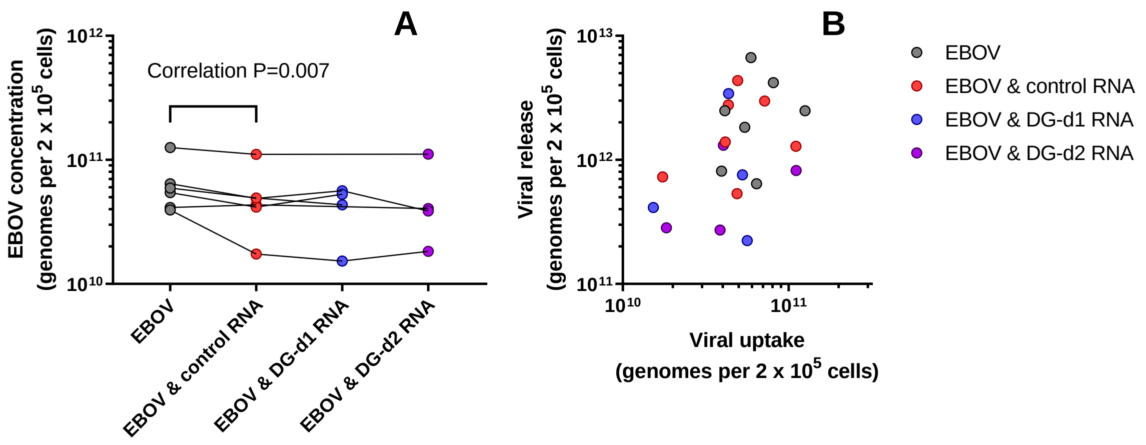

Supplemental figure 3: Establishing the initial conditions for the co-infection experiments. 2×10^5 Vero C1008 cells were infected with 1×10^6 $TCID_{50}$ infectious units of EBOV strain Ecran ($\sim 1 \times 10^{12}$ genomes) for 2 h then washed. The cells were then transfected with 1 µg of control RNA (red dots) or DG-d1 RNA (blue dots) or DG-d2 RNA (purple dots) for 4 h. Cells untreated with RNA and infected by Ebola virus (grey dots) was used for comparison. Cell samples were washed and taken. These were analysed for viral uptake (panel **A**). The connecting lines denote the six experiments performed and suggest a relationship between viral uptake and individual experiment. A Pearson's correlation was observed between the two controls (P=0.007). To understand if these differences affected viral output, the viral uptake was then compared to the virus release from cells exposed to identical conditions then left for 48 h and the supernatant samples analysed for viral titres (panel **B**). We found no strong evidence for this (R=0.388, P=0.067). Each data point on these graphs was generated as the geometric mean of 3 replicates generated from independent wells within the same experiment.
